# Supplementary material for: Cancer in children born after frozen-thawed embryo transfer: A cohort study
Source: PLoS Med. 2022 Sep 1;19(9):e1004078. doi: 10.1371/journal.pmed.1004078 (PMC9436139; doi:10.1371/journal.pmed.1004078)
Supplement: S1 Text — (DOCX) [file pmed.1004078.s002.docx]

**S1 Text. Prospective analysis plan:** Cancer in Children Born after Frozen-Thawed Embryo Transfer: A Cohort Study

**Research protocol / CoNARTaS/ Risk of cancer in ART conceived children.**

Nona Sargisian

Ulla-Britt Wennerholm

Christina Bergh

Birgitta Lannering

Max Petzold

and CoNARTaS group

2020.04.28,

**Research question**

Do children conceived after assisted reproductive technology (ART) have an increased risk of cancer?

**Background**

The increasing number of children born after use of assisted reproductive technology (ART) makes safety aspects of children born after ART important, both short- and long-term. Cancer in children is one outcome where studies so far have shown conflicting results.

In a large British cohort study (n=106 013 ART children) (Williams et al, 2013) no overall increased risk for cancer was found in the main analysis (standardized incidence ratio [SIR], 0.98; 95% confidence interval [CI], 0.81 to 1.19; P=0.87), while a slightly increased risk for cancer regarding hepatoblastoma (SIR, 3.64; 95% CI, 1.34 to 7.93; P=0.02) and rhabdomyosarcoma (SIR, 2.62; 95% CI, 1.26 to 4.82; P=0.02) was found in sub-analysis, however, based on few cases. In a retrospective Nordic population-based cohort study (n=91 796) (Sundh et al, 2014) there was neither any increased risk in overall cancer found (adjusted HR 1.08, 95% CI 0.91-1.27), nor in the group of leukaemia (adjusted hazard ratio [aHR] 1.06, 95% CI 0.80-1.41), one of the largest subgroups of childhood cancer. In subgroup analyses, an increased rate was found in 2 of 12 groups, central nervous system tumours (adjusted HR 1.44; 95% CI 1.01-2.05) and malignant epithelial neoplasms (adjusted HR 2.03; 95% CI 1.06-3.89). In a recent large population-based study from US (n=275 686) (Spector et al^[[1]](#endnote-1)^) a marginally significant association between IVF and overall childhood cancer was found (251.9 for IVF group and 192.7 for non-IVF group per 1000 000 person-year: HR 1.17; 95% CI, 1.00-1.63). In the subgroup of hepatic tumours, the increase was significant (HR 2,46; 95% CI 1.29-4.70).

In a Dutch study (Spaan et al) (n=24 269), including also a subfertile control group not undergoing IVF, the overall cancer risk was not increased in the ART population compared to the general population, neither in comparison with spontaneously conceived children from subfertile women. In a recent Danish cohort study, Hargreave et al found no significant association between fertility treatment and the risk of any type of cancer (HR 1.02, 95% CI; 0.91-1.15), or for any type of ART (HR 1.20, CI 95% CI; 0.96-1.49). There was a significant increased risk of cancer in children born after frozen embryo transfer compared to children born to fertile women (HR 2.43, 95% CI; 1.44-4.11), however based on only 14 children.

A recent systematic review of 18 cohort and 15 case-control studies by Chiavarini et al showed that the overall cancer risk for ART children was significantly increased (OR 1.34, 95% CI 1.22-1.47). Another systematic review by Wang et al reported a possible association between fertility treatment and higher risk of cancer (RR 1.16, 95% CI 1.01-1.32) but the researchers couldn’t show if the association depended on infertility per se or ART specifically. The above systematic reviews do, however, not include the studies by Spector et al, Spaan et al, and Hargreave et al.

**Study population**

The Committee of Nordic ART and Safety (CoNARTaS) was established 2008 for research focusing on ART pregnancies, perinatal outcomes as well aslong-term health of ART children and maternal outcomes (Opdahl et al, 2019). The database is located at Statistics Denmark in Copenhagen and currently includes all children born after ART and their mothers and all children born after spontaneous conception and their mothers, from start of IVF until 2014-2015.

In the present study we will use data from CoNARTaS database which includes crosslinking of ART children with several nationwide health data registries and national quality registries,thus comparing ART offspring with children born after spontaneous conception between 1994 and 2014 in Denmark, October 1990 and 2014 in Finland, 1984 and 2015 in Norway and 1985 and 2015 in Sweden. There are in total 172 161 ART children (including 127 376 singletons and 42 721 twins) and 7 681 797 spontaneously conceived children in the cohort. Inclusion criteria were all singletons, twins and high order multiples born alive after ART and spontaneous conception during the study period. Follow-up time for outcome of interest will time from birth until child’s age at diagnosis of first occurred cancer, emigration, death, 18^th^ birthday or end of a follow-up period (2014 for Finland, 2015 for Norway and Sweden and 2018 for Denmark).

**Data sources**

The following national data registries from Sweden, Denmark, Norway and Finland will be used:

Sweden: National ART registries, Medical Birth Registry (MBR), National Patient Register (NPR), Cause of Death Registry, Cancer Registry(1958-2015), Swedish Childhood Cancer Registry (national quality register), Statistics Sweden (SCB; for data on educational level and emigration)

Denmark: National ART registry, MBR, NPR, Cancer Registry (1943-2018),

Norway: MBR (including ART), NPR, Cancer Registry (1953-2015)

Finland: MBR (including ART), NPR, Cancer Registry (1953-2014)

Ethical approval was gained according to the actual legislation in each country for collecting data. Ethical approval in order to initiate a registry-based study is required in Sweden, which is obtained from Ethical Committee in Gothenburg (Dnr 214-12, T422-12, T516-15, T233-16, T300-17, T1144-17, T121-18, T1071-18, 2019-02347). Also, in Norway an approval given by the Regional Committee for medical and Health to proceed registry-based study (REK-Nord, 2010/1909) was obtained.

There is no requirement for ethical approval for registry based studies in Denmark and Finland.

**Cancer diagnosis**

Cancer diagnosis will be categorized into 12 main groups according to the International Classification of Childhood Cancer and International Classification of Diseases (ICD) codes: I. leukemias, II. lymphomas, III. CNS tumours, IV. neuroblastomas and peripheral nervous cell tumours, V. retinoblastomas, VI. renal tumours, VII. hepatic tumours, VIII. malignant bone tumours, IX. soft tissue and other extraosseous sarcomas, X. germ cells tumours, XI. other malignant epithelial and neoplasms and malignant melanomas, XII. other unspecified malignant neoplasms. Stratification will be made by origin of country but also by mode of conception.

| I. leukemias | C91-C96 |
| --- | --- |
| II. lymphomas | C81-C85 |
| III. CNS tumours | C70-C72, C751, C752, C753 |
| IV. Neuroblastomas and peripheral nervous cell tumours | C47, C74 |
| V. Retinoblastomas | C69 |
| VI. Renal tumours | C64-C68 |
| VII. Hepatic tumours | C23, C24 |
| VIII. Malignant bone tumours | C40-V41 |
| IX. Soft tissue and other extraosseous sarcomas | C46, C49 |
| X. Germ cells tumours | C54-C63 |
| XI. Other malignant epithelial neoplasms | C43-C44, C51-C53, C73, C75 |
| XII Other unspecified malignant neoplasms. | C00-C21, C22, C25, C30-39, C45, C50, C754, C755, C758, C759, C76, C80 |

**Statistical analysis**

Descriptive statistics will be given by numbers and percentages. We will use Cox proportional hazards models to estimate the risk of any cancer, with age as the time scale. We will compute each child´s time at risk from date of birth until whichever event occurred first: diagnosis of any cancer, emigration (available through 2014 for Denmark, through 2015 for Sweden and Norway, and not available for Finland), death (available through 2014 for Denmark and Finland, and 2015 for Norway and Sweden), 18^th^ birthday, or end of the follow-up period (December 31, 2014 for Finland, December 31, 2015 for Norway and Sweden, and December 31, 2018 for Denmark).

We will estimate crude and adjusted hazard ratios (HRs) with 95% confidence intervals (CIs). Adjustment will be done for the following confounders:

i. maternal/paternal confounders: maternal and paternal age, body mass index (BMI), maternal smoking, maternal education, parity, cause of infertility, singleton vs multiple birth.

ii. sex, major birth defects.

**Outcomes**

Main outcome of interest is overall cancer in ART children in comparison to cancer in spontaneously conceived children. Further separate analyses of subgroups of malignancies (leukaemia, brain tumour etc) will be performed. Subgroup analyses for IVF, FET (frozen embryo transfers) will also be performed.

**Modifications to the original analysis plan,** 2021.10.03

The following modifications have been made from the initial study plan:

- After consideration we chose classification of childhood cancer by The International Classification of Childhood Cancer (ICCC-3). According to ICCC-3 cancer diagnoses are divided into 12 groups by the morphology code, the topographic code and also according to the behaviour of the tumour ie benign-malignant (Steliarova-Foucher et al., 2018). This classification enables identification of malignant tumours with the exception from central nervous system tumours.
- The increasing number in FET cycles worldwide and findings in Hargreave et al. 2019 concerning FET and cancer, made us, in addition to any ART focus on FET and risk ofcancer.
- Recent study showed that children with birth defects in ART group had a greater risk of developing cancer compared to children born after spontaneous conception. (Luke et al., 2020). Data on birth defects as variable of interest was obtained from relevant registries. Additional adjustment for major birth defects was made.
- Country of birth for the child and calendar year of birth were added as confounders and included in the adjusted analyses. Variables such as paternal age and maternal BMI were excluded since these variables were either not possible to obtain from all countries or had a lot of missing values.
- Since macrosomia and LGA are known risk factors for childhood cancer (Magnusson et al., 2021) adjustments were performed for macrosomia (birthweight >4000g) and birthweight as a continuous variable, performed in separate models.
- Sensitivity analysis was performed for maternal smoking during pregnancy (yes/no) and maternal highest educational level achieved during the study period (low, medium, high) as a covariate.

**References**

Williams CL, Bunch KJ, Stiller CA, Murphy MF, Botting BJ, Wallace WH, et al. Cancer risk among children born after assisted conception. N Engl J Med. 2013 Nov 7;369(19):1819-27. doi: 10.1056/NEJMoa1301675. PMID: 24195549.

Sundh KJ, Henningsen AK, Källen K, Bergh C, Romundstad LB, Gissler M, et al. Cancer in children and young adults born after assisted reproductive technology: a Nordic cohort study from the Committee of Nordic ART and Safety (CoNARTaS). Hum Reprod. 2014 Sep;29(9):2050-7. doi: 10.1093/humrep/deu143. Epub 2014 Jul 2. PMID: 24990274.

Spector LG, Brown MB, Wantman E, Letterie GS, Toner JP, Doody K, Ginsburg E, et al. Association of In Vitro Fertilization With Childhood Cancer in the United States. JAMA Pediatr. 2019 Jun 1;173(6):e190392. doi: 10.1001/jamapediatrics.2019.0392. Epub 2019 Jun 3. PMID: 30933244; PMCID: PMC6547076.

Spaan M, van den Belt-Dusebout AW, van den Heuvel-Eibrink MM, Hauptmann M, Lambalk CB, Burger CW, et al. Risk of cancer in children and young adults conceived by assisted reproductive technology. Hum Reprod. 2019 Apr 1;34(4):740-750. doi: 10.1093/humrep/dey394. PMID: 30715305; PMCID: PMC6443110.

Hargreave M, Jensen A, Hansen MK, Dehlendorff C, Winther JF, Schmiegelow K, et al. Association Between Fertility Treatment and Cancer Risk in Children. JAMA. 2019 Dec 10;322(22):2203-2210. doi: 10.1001/jama.2019.18037. PMID: 31821431; PMCID: PMC7081748.

Chiavarini, M Ostorero A, Naldini G, Faiani R., et al. Cancer risk in children and young adults (offsping) born after medically assisted reproduction: a systematic review and meta-analysis. J Multidisciplinary Scientific Journal. 2019, 2(4), 430-448; https://doi.org/10.3390/j2040028

Steliarova-Foucher E. International Classification of Childhood Cancer, In Press. In: Cancer IAfRo, ed. Lyon 2021.

Opdahl S, Henningsen AA, Bergh C, Gissler M, Romundstad LB, Petzold M, et al. Data Resource Profile: Committee of Nordic Assisted Reproductive Technology and Safety (CoNARTaS) cohort. Int J Epidemiol. 2020 Apr 1;49(2):365-366f. doi: 10.1093/ije/dyz228. PMID: 31742613.

Wang T, Chen L, Yang T, Wang L, Zhao L, Zhang S, Ye Z, Chen L, Zheng Z, Qin J. Cancer risk among children conceived by fertility treatment. Int J Cancer. 2019 Jun 15;144(12):3001-3013. doi: 10.1002/ijc.32062. Epub 2019 Jan 3. PMID: 30548591; PMCID: PMC6590158.

Luke B, Brown MB, Nichols HB, Schymura MJ, Browne ML, Fisher SC, et al. Assessment of Birth Defects and Cancer Risk in Children Conceived via In Vitro Fertilization in the US. JAMA Netw Open. 2020 Oct 1;3(10):e2022927. doi: 10.1001/jamanetworkopen.2020.22927. Erratum in: JAMA Netw Open. 2020 Dec 1;3(12):e2031636. PMID: 33119107; PMCID: PMC7596575.

Magnusson Å, Laivuori H, Loft A, Oldereid NB, Pinborg A, Petzold M, et al. The Association Between High Birth Weight and Long-Term Outcomes-Implications for Assisted Reproductive Technologies: A Systematic Review and Meta-Analysis. Front Pediatr. 2021 Jun 23;9:675775. doi: 10.3389/fped.2021.675775. PMID: 34249812; PMCID: PMC8260985.

1. [↑](#endnote-ref-1)
