# Supplementary figures and images for: Cancer in children born after frozen-thawed embryo transfer: A cohort study
Source: PLoS Med. 2022 Sep 1;19(9):e1004078. doi: 10.1371/journal.pmed.1004078 (PMC9436139; doi:10.1371/journal.pmed.1004078)

**S1 Fig.**

**
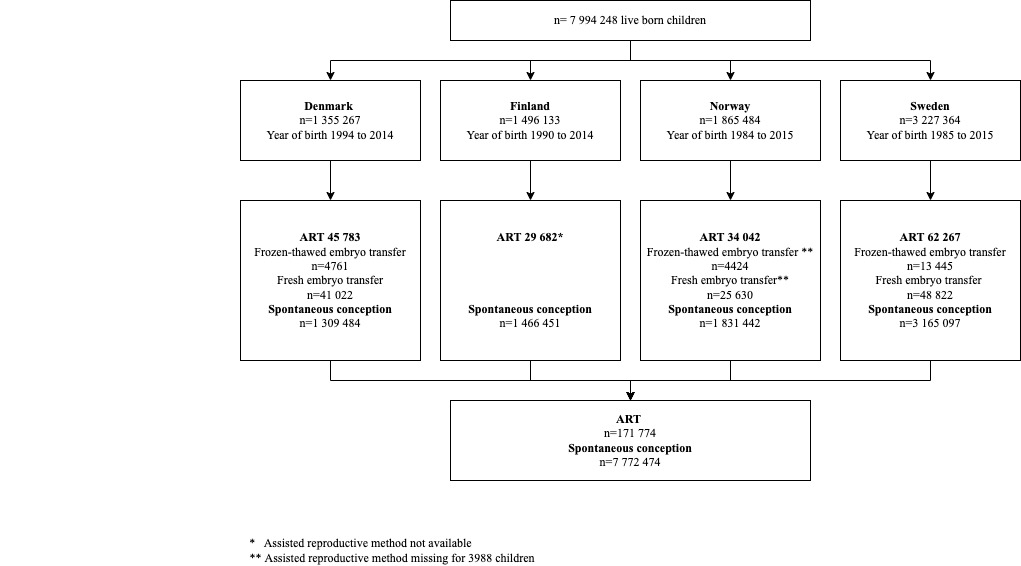
**

Supplement: S1 Fig — (DOCX) [file pmed.1004078.s003.docx]
