# Supplementary material for: Cancer in children born after frozen-thawed embryo transfer: A cohort study
Source: PLoS Med. 2022 Sep 1;19(9):e1004078. doi: 10.1371/journal.pmed.1004078 (PMC9436139; doi:10.1371/journal.pmed.1004078)

**S2 Fig.** Hazard ratios with 95% confidence interval for independent covariates including major birth defects for risk of cancer in children born after frozen-thawed embryo transfer versus fresh embryo transfer and versus spontaneous conception


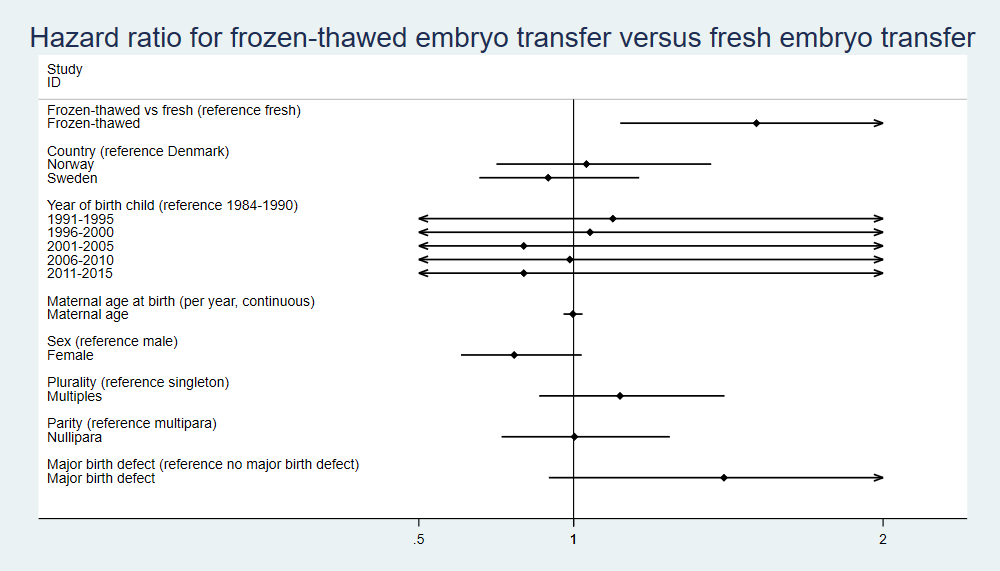


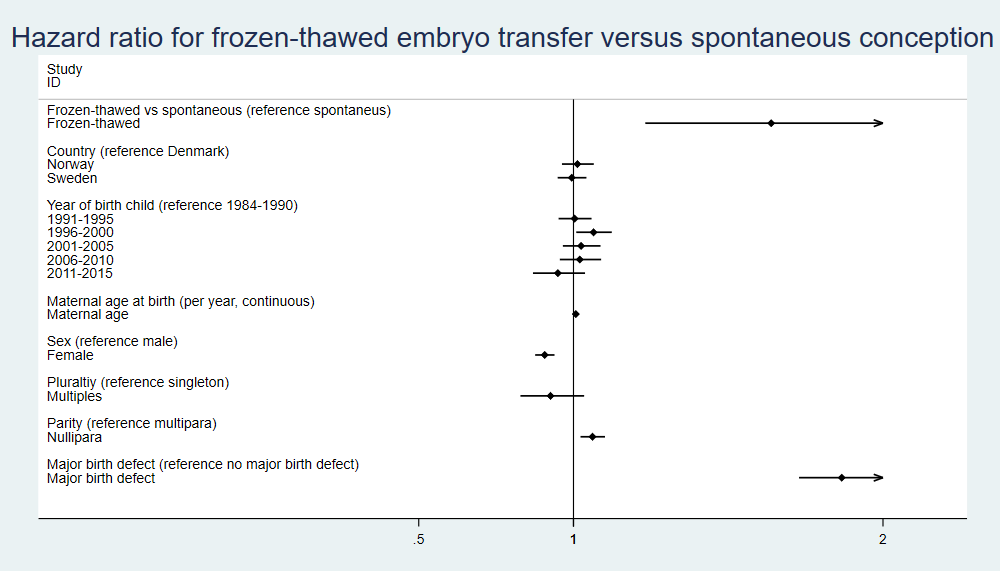

Supplement: S2 Fig — (DOCX) [file pmed.1004078.s004.docx]
