# Supplementary material for: Cancer in children born after frozen-thawed embryo transfer: A cohort study
Source: PLoS Med. 2022 Sep 1;19(9):e1004078. doi: 10.1371/journal.pmed.1004078 (PMC9436139; doi:10.1371/journal.pmed.1004078)
Supplement: S1 Table — (DOCX) [file pmed.1004078.s005.docx]

**S1 Table.** Data sources and standards

|  | Denmark | Finland | Norway | Sweden |
| --- | --- | --- | --- | --- |
| Birth |  |  |  |  |
| Sources | MBR | MBR | MBR | MBR |
| Standards | Responsible physician at delivery^1^ | Responsible physician at delivery^1^ | Responsible physician at delivery^1^ | Responsible physician at delivery^1^ |
| ART |  |  |  |  |
| Sources | ART Registry | MBR | MBR | ART Registry |
| Standards | Responsible physcian^1^ | IVF units/patients^2^ | IVF units^3^ | Responsible physcian^1^ |
| Birth defects |  |  |  |  |
| Sources | National Patient Registry | MBR+Registry of Birth defects | MBR | MBR+ National Patient Registry |
| Standards | Responsible physcian^1^ | Responsible physcian^1^ | Responsible physcian^1^ | Responsible physcian^1^ |
| Cancer |  |  |  |  |
| Sources | Cancer Registry | Cancer Registry | Cancer Registry | Cancer Registry |
| Standards | Responsible physcian^1^ | Responsible physcian^1^ | Responsible physcian^1^ | Responsible physcian^1^ |

MBR; Medical Birth Registry, ART; Assisted Reproductive Technology ^1^reported by responsible physician to the registry using standardized definition ^2^public funded IVF units report to MBR, patients from private units report at delivery ^3^all ongoing pregnancies are reported in gestational week 7-8 from the IVF units to MBR
