# Supplementary material for: Cancer in children born after frozen-thawed embryo transfer: A cohort study
Source: PLoS Med. 2022 Sep 1;19(9):e1004078. doi: 10.1371/journal.pmed.1004078 (PMC9436139; doi:10.1371/journal.pmed.1004078)
Supplement: S2 Table — (DOCX) [file pmed.1004078.s006.docx]

**S2 Table.** Main classification of cancer diagnosis groups according to the International Classification of Childhood Cancer (ICCC-3).^1^

| **Diagnostic group** |
| --- |
| I. Leukemias, myeloproliferative diseases, and myelodysplastic diseases |
| II. Lymphomas and reticuloendothelial neoplasms |
| III. CNS and miscellaneous intracranial and intraspinal neoplasms^a^ |
| IV. Neuroblastoma and other peripheral nervous cell tumors |
| V. Retinoblastoma |
| VI. Renal tumors |
| VII. Hepatic tumors |
| VIII. Malignant bone tumors |
| IX. Soft tissue and other extraosseous sarcomas |
| X. Germ cell tumors, trophoblastic tumors, and neoplasms of gonads^b^ |
| XI. Other malignant epithelial neoplasms and malignant melanomas |
| XII. Other and unspecified malignant neoplasms |

CNS; central nervous system

^a^CNS tumors with nonmalignant behavior are included

^b^intracranial and intraspinal germ cells tumors with nonmalignant behavior are included

**Reference**

1. U.S. Department of Health and Human Services. National Institutes of Health. National Cancer Institute. International Classification of Childhood Cancer. ICCC Recode Third Edition ICD-O-3/IARC2017. Available from: <https://seer.cancer.gov/iccc/iccc-iarc-2017.html> (Accessed May 16, 2022)
