# Supplementary material for: Cancer in children born after frozen-thawed embryo transfer: A cohort study
Source: PLoS Med. 2022 Sep 1;19(9):e1004078. doi: 10.1371/journal.pmed.1004078 (PMC9436139; doi:10.1371/journal.pmed.1004078)
Supplement: S3 Table — (DOCX) [file pmed.1004078.s007.docx]

**S3 Table.** Characteristics of study population by mode of conception defined as frozen-thawed embryo transfer, fresh embryo transfer, or spontaneous conception in children born in Denmark 1994-2014, Norway 1984-2015, or Sweden 1985-2015^a^

|  | **Frozen-thawed embryo transfer**  **N=22 630** | **Fresh embryo transfer**  **N=115 474** | **Spontaneous conception**  **N=6 306 023** |
| --- | --- | --- | --- |
| Child characteristics |  |  |  |
| *Calendar year at birth, N (%)* |  |  |  |
| 1984–1990 | 32  (0.1) | 1,437  (1.2) | 1,030,650  (16.3) |
| 1991–1995 | 634  (2.8) | 7861  (6.8) | 1 000 138  (15.9) |
| 1996–2000 | 1567  (6.9) | 19 121  (16.6) | 1 050 430  (16.7) |
| 2001–2005 | 2771  (12.2) | 26 326  (22.8) | 1 056 015  (16.8) |
| 2006–2010 | 7 097  (31.4) | 30 632  (26.5) | 1 122 293  (17.8) |
| 2011–2015 | 10 529  (46.5) | 30 097  (26.1) | 1 046 497  (16.6) |
| *Birthweight, N (%)* |  |  |  |
| Very low birthweight, <1500 g | 430  (1.9) | 3783  (3.3) | 46 751  (0.8) |
| Low birthweight,  <2500 g | 1834  (8.1) | 15 830  (13.8) | 225 324  (3.6) |
| $\boldsymbol{Makrosomia \geq}$4000 g | 4390  (19.5) | 12 146  (10.6) | 1 186 593  (18.9) |
| Birthweight, g,  mean (SD) | 3410  (725) | 3151  (753) | 3515  (588) |
| Missing data for birthweight, N (%) | 80  (0.4) | 659  (0.6) | 33 117  (0.4) |
| *Gestational age, N (%)* |  |  |  |
| Extremely preterm birth , <28+0 weeks | 190  (0.8) | 1502  (1.3) | 18 336  (0.3) |
| Very preterm birth, <32+0 weeks | 402  (1.8) | 3179  (2.8) | 40 260  (0.7) |
| Preterm birth, <37+0 weeks | 2604  (11.5) | 18 990  (16.5) | 353 911  (5.7) |
| Postterm birth, ≥42+0 weeks | 977  (4.3) | 3387  (2.9) | 333 831  (5.4) |
| Gestational age, days, mean (SD) | 273  (18) | 270  (20) | 278  (14) |
| Missing data for gestational age, N (%) | 42  (0.2) | 371  (0.3) | 122 688  (1.6) |
|  |  |  |  |
|  |  |  |  |
|  |  |  |  |
| *Plurality, N (%)* |  |  |  |
| Singleton | 18 872  (83.4) | 83 623  (72.4) | 6 142 874  (97.4) |
| Multiples | 3758  (16.6) | 31 851  (27.6) | 163 149  (2.6) |
| *Birth defects, N (%)* |  |  |  |
| Any major defects^c^ (non-chromosomal or chromosomal defects) | 1003  (4.4) | 5598  (4.8) | 196 547  (3.1) |
| Major birth defects^c^  (non-chromosomal) | 969  (4.3) | 5421  (4.7) | 191 234  (3.0) |
| Chromosomal defects (with or without other major birth defects^c^) | 34  (0.15) | 177  (0.15) | 5313  (0.08) |
| Male sex, N (%) | 11 567  (51.4) | 59 048  (51.1) | 3 239 628  (51.4) |
| Age at cancer diagnosis (year) mean (SD), median (range) | 4.5 (4.0)  3.0 (0-18) | 6.1 (5.1)  4.5 (0-18) | 6.9 (5.4)  5.3 (0-18) |
| Follow-up time, (year),  mean (SD), median (range) | 7.1 (5.1)  6.0 (0-18) | 10.5 (5.7)  10.4 (0-18) | 12.7 (5.9)  15.0 (0-18) |
| Maternal characteristics |  |  |  |
| Age at delivery, (year), mean (SD) | 34.4 (4.1) | 33.8 (4.1) | 29.7 (5.1) |
| *Parity, N (%)* |  |  |  |
| Primiparous | 12 544  (55.6) | 81 203  (70.6) | 2 651 362  (42.2) |
| Smoking during pregnancy *^d^*, N (%) | 797  (3.7) | 7202  (6.9) | 764 292  (14.9) |
| Missing data for smoking, N (%) | 1203  (5.3) | 10 540  (9.1) | 1 176 347  (15.1) |
| BMI (kg/m^2^), mean (SD) | 24.2  (4.0) | 24.3  (4.1) | 24.1  (4.5) |
| Missing data for BMI, N (%) | 4850  (21.4) | 41 913  (36.3) | 2 948 544  (37.9) |
| *Highest educational level, N (%)^e,f^* |  |  |  |
| Low (ISCED <5) | 7328  (40.3) | 43 196  (48.1) | 2 498 872  (55.8) |
| Medium (ISCED 5-6) | 6070  (33.3) | 27 903  (31.1) | 1 185 345  (26.5) |
| High (ISCED 7-8) | 4011  (22.0) | 15 863  (17.7) | 544 551  (12.2) |
| Missing data for educational level,  N (%) | 797  (4.4) | 2882  (3.1) | 245 813  (5.5) |
| *Assisted reproduction method, N (%)* |  |  |  |
| IVF | 12 393  (54.8) | 68 363  (59.2) | - |
| ICSI | 7967  (35.2) | 46 719  (40.5) | - |
| Missing data for IVF/ICSI | 2270  (10.0) | 392  (0.3) | - |
| Cleavage stage embryo | 18 573  (82.1) | 111 493  (96.6) |  |
| Blastocysts | 3385  (15.0) | 3468  (3.0) |  |
| Missing data for embryo stage | 672  (3.0) | 513  (0.4) |  |

BMI; body mass index, ICSI; intracytoplasmic sperm injection, ISCED; international standard classification of education, IVF; in vitro fertilization, LGA; large for gestational age, SGA; small for gestational age

^a^Children born in Denmark, Norway, and Sweden because no data are available from Finland on frozen/fresh embryo transfer

^c^Major birth defects defined according to the EUROCAT classification system^1^

^d^Data for Denmark, Sweden but only birth cohorts since 1999 from Norway when smoking habits were first registered

^e^Data calculated for Denmark and Sweden because no data were available for Norway

^f^Educational level according to International Standard Classification of Education (ISCED2011), ISCED <5=primary, secondary or post-secondary non tertiary education, ISCED 5-6=first stage of tertiary education (bachelors or equivalent), ISCED 7-8=second stage of tertiary education (master, doctorate, or more)^2^

**References**

1. EUROCAT. EUROCAT Guide 1.4 Online. Available online at: <http://www.eurocat> network.eu/aboutus/datacollection/guidelinesforregistration/ guide1_4
2. <https://ec.europa.eu/eurostat/statistics-explained/index.php?title=International_Standard_Classification_of_Education_(ISCED)> (Accessed, November1, 2021)
