# Supplementary material for: Cancer in children born after frozen-thawed embryo transfer: A cohort study
Source: PLoS Med. 2022 Sep 1;19(9):e1004078. doi: 10.1371/journal.pmed.1004078 (PMC9436139; doi:10.1371/journal.pmed.1004078)
Supplement: S4 Table — (DOCX) [file pmed.1004078.s008.docx]

**_S4 Table._** _Incidence rate of overall cancer and type of cancer according to International Classification of Childhood Cancer (ICCC-3)_^1^ _categories before 18 years of age by first diagnosis and country of birth in children born in Denmark, Finland, Norway, or Sweden between 1984 and 2015._

| Cancer Type  (ICCC-3 category)^1^ | All countries  N=7 944 248 children  N=98 732 823 person-years | | | Denmark  N=1 355 267 children  N=17 780 806 person-years | | | Finland  N=1 496 133 children  N=17 403 330 person-years | | | Norway  N=1 865 484 children  N=23 680 407 person-years | | | Sweden  N=3 227 364 children  N=39 868 280 person-years | | |
| --- | --- | --- | --- | --- | --- | --- | --- | --- | --- | --- | --- | --- | --- | --- | --- |
|  | No. of children with cancer | Incidence rate | | No. of children with cancer | Incidence rate | | No. of children with cancer | Incidence rate | | No. of children with cancer | Incidence rate | | No. of  children with cancer | Incidence rate | |
|  |  | Per  1000  children | Per  100 000 person- years |  | Per  1000  children | Per  100 000 person- years |  | Per  1000  children | Per  100 000 person- years |  | Per  1000  children | Per  100 000 person- years |  | Per  1000  children | Per  100 000 person- years |
| Any cancer (I-XII) | 16 512 | 2.08 | 16.72 | 2949 | 2.18 | 16.58 | 3189 | 2.13 | 18.32 | 3904 | 2.09 | 16.49 | 6472 | 2.01 | 16.23 |
| Leukemia (I) | 5032 | 0.63 | 5.10 | 925 | 0.68 | 5.20 | 981 | 0.66 | 5.64 | 1149 | 0.62 | 4.85 | 1977 | 0.61 | 4.96 |
| Lymphomas (II) | 1729 | 0.22 | 1.75 | 366 | 0.27 | 2.06 | 342 | 0.23 | 1.97 | 451 | 0.24 | 1.90 | 570 | 0.18 | 1.43 |
| CNS tumors (III) | 4167 | 0.52 | 4.22 | 602 | 0.44 | 3.38 | 782 | 0.52 | 4.49 | 1061 | 0.57 | 4.48 | 1722 | 0.53 | 4.32 |
| Neuroblastoma and other peripheral nervous cell tumors (IV) | 945 | 0.12 | 0.96 | 158 | 0.12 | 0.89 | 208 | 0.14 | 1.20 | 212 | 0.11 | 0.90 | 367 | 0.11 | 0.92 |
| Retinoblastoma (V) | 407 | 0.05 | 0.41 | 89 | 0.07 | 0.50 | 71 | 0.05 | 0.40 | 99 | 0.05 | 0.42 | 148 | 0.05 | 0.37 |
| Renal tumors (VI) | 858 | 0.11 | 0.87 | 128 | 0.09 | 0.72 | 171 | 0.11 | 0.98 | 172 | 0.09 | 0.73 | 387 | 0.12 | 0.97 |
| Hepatic tumors (VII) | 232 | 0.03 | 0.23 | 39 | 0.03 | 0.22 | 35 | 0.02 | 0.20 | 63 | 0.03 | 0.27 | 95 | 0.03 | 0.23 |
| Bone tumors (VIII) | 654 | 0.08 | 0.66 | 134 | 0.10 | 0.75 | 89 | 0.06 | 0.51 | 150 | 0.08 | 0.63 | 281 | 0.09 | 0.70 |
| Soft tissue sarcomas (IX) | 893 | 0.11 | 0.90 | 159 | 0.12 | 0.89 | 169 | 0.11 | 0.97 | 212 | 0.11 | 0.90 | 353 | 0.11 | 0.89 |
| Germ cell and gonadal tumors (X) | 674 | 0.08 | 0.68 | 121 | 0.10 | 0.68 | 116 | 0.08 | 0.67 | 181 | 0.10 | 0.76 | 256 | 0.08 | 0.64 |
| Epithelial tumors and melanoma (XI) | 834 | 0.10 | 0.84 | 219 | 0.16 | 1.23 | 205 | 0.14 | 1.18 | 150 | 0.08 | 0.63 | 256 | 0.08 | 0.64 |
| Other and unspecified tumors (XII) | 88 | 0.01 | 0.09 | 9 | 0.007 | 0.05 | 19 | 0.01 | 0.11 | 4 | 0.002 | 0.02 | 56 | 0.02 | 0.14 |

CNS; central nervous system
