# Supplementary material for: Cancer in children born after frozen-thawed embryo transfer: A cohort study
Source: PLoS Med. 2022 Sep 1;19(9):e1004078. doi: 10.1371/journal.pmed.1004078 (PMC9436139; doi:10.1371/journal.pmed.1004078)
Supplement: S5 Table — (DOCX) [file pmed.1004078.s009.docx]

**_S5 Table._** _Incidence rate and risk of specific type of cancer according to International Classification of Childhood Cancer (ICCC-3)_^1^ _by first diagnosis before 18 years of age in children born after frozen-thawed embryo transfer, fresh embryo transfer or spontaneous conception in Denmark 1994-2014, Norway 1984-2015, or Sweden 1985-2015_

| Cancer type  (ICCC-3 categories)^1^ | Frozen-thawed embryo transfer  N=22 630  N=159 566 person-years | | | Fresh embryo transfer  N=115 474  N=1 207 598 person-years | | | Spontaneous conception  N=6 306 023  N=79 920 380 person-years | | | Frozen-thawed embryo transfer vs  fresh embryo transfer | | Frozen-thawed embryo transfer vs  spontaneous conception | |
| --- | --- | --- | --- | --- | --- | --- | --- | --- | --- | --- | --- | --- | --- |
|  | No. of children with cancer | Incidence rate | | No. of children with cancer | Incidence rate | | No. of children with cancer | Incidence rate | | Crude HR  (95% CI)  *p*-value | Adjusted HR^a^  (95% CI)  *p*-value | Crude HR  (95% CI)  *p*-value | Adjusted HR^a^  (95% CI)  *p*-value |
|  |  | Per  1000  children | Per  100 000  person- years |  | Per  1000  children | Per  100 000  person- years |  | Per  1000  children | Per  100 000  person- years |  |  |  |  |
| Leukemia (I) | 23 | 1.02 | 14.41 | 75 | 0.65 | 6.21 | 3952 | 0.63 | 4.94 | 2.01  (1.26 to 3.22)  0.004 | 2.25  (1.38 to 3.68)^b^  0.001 | 2.41  (1.60 to 3.64)  <0.001 | 2.22  (1.47 to 3.35)^c^  <0.001 |
| Lymphomas (II) | 4 | 0.18 | 2.51 | 21 | 0.18 | 1.74 | 1361 | 0.22 | 1.70 | NA^d^ | NA^d^ | NA^d^ | NA^d^ |
| CNS tumor  (III) | 10 | 0.44 | 6.27 | 59 | 0.51 | 4.89 | 3314 | 0.53 | 4.15 | 1.28  (0.65 to 2.51)  0.47 | 1.21  (0.60 to 2.41)  0.60 | 1.45  (0.78 to 2.71)  0.24 | 1.49  (0.80 to 2.78)  0.21 |
| Neuroblastoma and other peripheral nervous cell tumors (IV) | <3^e^ | <0.13^e^ | <1.9^e^ | 6 | 0.05 | 0.50 | 729 | 0.12 | 0.91 | NA^d^ | NA^d^ | NA^d^ | NA^d^ |
| Retinoblastoma (V) | <3^e^ | <0.13^e^ | <1.9^e^ | <3^e^ | <0.03^e^ | <0.25^e^ | 333 | 0.05 | 0.42 | NA^d^ | NA^d^ | NA^d^ | NA^d^ |
| Renal tumors (VI) | <3^e^ | <0.13^e^ | <1.9^e^ | 12 | 0.10 | 0.99 | 673 | 0.11 | 0.84 | NA^d^ | NA^d^ | NA^d^ | NA^d^ |
| Hepatic tumors (VII) | <3^e^ | <0.13^e^ | <1.9^e^ | 5 | 0.04 | 0.41 | 190 | 0.03 | 0.24 | NA^d^ | NA^d^ | NA^d^0.07 | NA^d^ |
| Bone tumors (VIII) | <3^e^ | <0.13^e^ | <1.9^e^ | <3^d^ | <0.03^e^ | <0.25^e^ | 562 | 0.09 | 0.70 | NA^d^ | NA^d^ | NA^d^ | NA^d^ |
| Soft tissue sarcomas (IX) | <3^e^ | <0.13 | <1.9^e^ | 21 | 0.18 | 1.74 | 701 | 0.11 | 0.88 | NA^d^ | NA^d^ | NA^d^ | NA^d^ |
| Germ cell and gonadal tumors (X) | 0 | - | - | 7 | 0.06 | 0.58 | 550 | 0.09 | 0.69 | NA^d^ | NA^d^ | NA^d^ | NA^d^ |
| Epithelial tumors and melanoma (XI) | 0 | - | - | 16 | 0.14 | 1.32 | 612 | 0.10 | 0.77 | NA^d^ | NA^d^ | NA^d^ | NA^d^ |
| Other and unspecified  tumors (XII) | 0 | - | - | <3^e^ | <0.03 | <0.25^e^ | 67 | 0.01 | 0.08 | NA^d^ | NA^d^ | NA^d^ | NA^d^ |

CI; confidence interval, CNS; central nervous system, HR; hazard ratio; NA; not applicable

^a^adjusted for sex, plurality, year of birth, country of birth, maternal age at birth, parity

**^b^**Leukemia: Frozen embryo transfer vs fresh embryo transfer: adjusted for sex, plurality, year of birth, country of birth, maternal age at birth, parity, and macrosomia: adjusted HR (aHR), 2.12 (95% CI, 1.30 to 3.48), *p*=0.003

**^b^**Leukemia: Frozen embryo transfer vs fresh embryo transfer: adjusted for sex, plurality, year of birth, country of birth, maternal age at birth, parity, and major birth defects: aHR, 2.26 (95% CI, 1.38 to 3.68), *p*=0.001

**^b^**Leukemia: Frozen embryo transfer vs fresh embryo transfer: adjusted for sex, plurality, year of birth, country of birth, maternal age at birth, parity, and embryo stage: aHR, 2.34 (95% CI 1.44 to 3.82), *p*=0.001

**^c^**Leukemia: Frozen embryo transfer vs spontaneous conception: adjusted for sex, plurality, year of birth, country of birth, maternal age at birth, parity, and macrosomia: aHR, 2.21 (95% CI, 1.47 to 3.35), *p*<0.001

**^c^**Leukemia: Frozen embryo transfer vs spontaneous conception: adjusted for sex, plurality, year of birth, country of birth, maternal age at birth, parity, and major birth defects: aHR, 2.20 (95% CI, 1.46 to 3.33), *p*<0.001

^d^Numbers too small (n <10 in ART group) to calculate a stable estimate

^e^These data are not reported as exact numbers to protect patient confidentiality
