# Supplementary material for: Cancer in children born after frozen-thawed embryo transfer: A cohort study
Source: PLoS Med. 2022 Sep 1;19(9):e1004078. doi: 10.1371/journal.pmed.1004078 (PMC9436139; doi:10.1371/journal.pmed.1004078)
Supplement: Attachment — Submitted filename: Cancer in Children After Frozen Embryo Transfer, Annals Int Med, December, 2021.docx [file pmed.1004078.s010.docx]

Review of Cancer in Children Born After Frozen-Thawed Embryo Transfer: A Cohort Study, by Sargisian et al.

Overall:

The authors present a detailed manuscript on the risk of childhood cancer by method of conception, using a population-based analysis spanning a 30-year period from four Nordic countries. This is an important area of research, as conception using assisted reproductive technology (ART) and in vitro fertilization (IVF) has risen dramatically worldwide since the first birth from this technology in 1978. This consortium of researchers (the CoNARTaS group [Committee of Nordic Assisted Reproductive Technology and Safety]) have been leaders in pooling population-based data to answer important questions about maternal and child health after assisted reproduction.

Upon deeper inspection, there are numerous gaps and inconsistencies in the data between the four countries in this study including the sources, types, and years reported. Although the authors refer the reader to other references for detail regarding the data from each country (References 24-30, and 42), it would be more relevant and transparent to include a table within this manuscript detailing the birth, ART, IVF, birth defects, and cancer data from each country, and the sources and standards for each type of data (self-reported by the mother at delivery versus by the physician or midwife, reported by the physician to a registry using standardized definitions). In addition, the data should be limited to residents of each country who received their ART or IVF treatment within their own country.

Limitations:

-The authors rely on several references (References 24-30 and 42, and the Marsál et al reference in the Supplemental Tables section) to describe important measures used in this study; this requires reading through those additional references to understand what was used. The birthweight reference for classifying infants as small-for-gestation (SGA) (Marsál et al, 1996), which is based on only 86 uncomplicated pregnancies, defines SGA as -2 SD below the mean, although the mean for each gestation (days) is not given; there is no definition for LGA in this reference, although the authors of this analysis reference Marsál et al for both SGA and LGA in their tables (Tables S2, S3). A preferable measure to define both SGA and LGA is birthweight z-score, with cutoffs of <1.28 and >1.28, respectively. There are several methods available to calculate birthweight z-scores, including the Fetal Medicine Foundation weight charts^[1]^ (which are also available online at <https://fetalmedicine.org/research/assess/bw>), and the US reference by Talge et al^[2]^, which is based on 7.8 million births in 2009-10 from 22-44 weeks gestation; means and standard deviations for birthweights by infant sex are given for each week, from which z-scores may be calculated.

Specific Comments:

-Throughout this manuscript, the authors state that the study population is based on births in 1984-2015 in four Nordic countries (including the sentence in the Methods section of the Abstract, “This cohort study included 171,774 children born after use of assisted reproductive technology (ART) and 7,772,474 children born after spontaneous conception, ***representing all children born between 1984 and 2015 in four Nordic countries***). In evaluating this statement, the study population actually includes 1994-2014 births in Denmark, 1990-2014 births in Finland, 1984-2015 births in Norway, and 1985-2015 births in Sweden.

-The description of the study populations by each country is given in Table S2; this description should have been the first table in this paper, and not put in the supplementary section. In addition, the racial and ethnic composition of the study population is not given, nor are these factors included in any of the regression models. This is an important limitation, as there are well-establish differences in childhood cancer by these two factors.

-The ART group represents a combination of both fresh and frozen embryo transfers, with cryopreservation reported for varying periods of time (1994-2014 for Denmark, 1988-2015 for Norway, 1990-2015 for Sweden), or not at all (Finland), and with cryopreservation methods (slow-freezing or vitrification) only reported for 2009-2014 in Denmark and 2006-2015 for Sweden; data on the use of ICSI is only available for 1994-2014 in Denmark, 1996-2015 in Norway, and 1992-2015 in Sweden; and data on culture duration (day of embryo transfer) only available for 1994-2014 in Denmark, 2011-2015 in Norway, and 1985-2015 in Sweden (Table 3 in Reference 24, Opdahl et al, Int J Epidemiology 2019; 1-8).

-The spontaneously-conceived births include women who were treated with non-IVF ART (ovulation induction and intrauterine insemination), resulting in some degree of misclassification. A new subgroup could be generated (non IVF-ART) for 2007-2015 births in Denmark, for 2016-17 births in Finland; it is unclear if this data is available in Norway and Sweden.

-Medical tourism is frequent, particularly in Denmark. ART conception is included in the Medical Birth Registry in Norway, based on information provided by the mother at delivery “…allowing for registration of children conceived after ART treatment abroad” [Opdahl et al, Int J Epidemiology 2020; 49:365-366f].

-The CoNARTaS description of birth defects registries (from Table 2 in Reference 24) in the four countries included in this analysis indicate that Finland has maintained this type of registry for 1990-2014 births, Sweden has done so for 1964-2015 births, while reporting birth defects has been through the Medical Birth Registry in Norway, and is not available for births in Denmark.

-Major birth defects should be separated into chromosomal, nonchromosomal, and the presence of both types within the same child, as it is well-established that children with chromosomal birth defects are at much greater risk for childhood cancer.

-Multifetal pregnancies are not divided into twins, triplets, and higher-order births; this should be specified.

-There are too few cancer cases to present models for frozen embryo transfer, and the models are missing critical factors such as infertility diagnoses, the use of assisted hatching and ICSI, the day of embryo transfer (days in culture, as cleavage-stage versus blastocyst-stage), as well as maternal race and ethnicity (as stated earlier). Most of the supplemental tables and models should be eliminated (Table 2 results of the 12 cancer types includes 4 models based on <10 cases of cancer in ART children and 8 models based on <30 cases among ART children).

-The authors minimize acknowledgement of the limitations of their study, citing that it is observational data, and that non-IVF ART (intrauterine insemination or ovulation induction) could not be excluded, but only accounted for a “very small proportion of the spontaneous conception cohort.” As stated above, this data is available for Denmark, and may account for a much larger proportion of births than stated. The Center for Disease Control estimated that non-IVF ART accounts for four-times as many IVF-conceived births on an annual basis.^[3]^

Minor Comments:

Spelling of:

New Zealand (line 72, page 4)

Macrosomia (Supplemental tables)

Carry over headings when tables cross multiple pages (Table S2)

1. Nicolaides KH, Wright D, Syngelaki A, Wright A, Akolekar R. Fetal Medicine Foundation fetal and neonatal population weight charts. Ultrasound in Obstetrics and Gynecology 2018, pp 1-8. Doi: 10.1002/uog.19073.
2. Talge NM, Mudd LM, Sikorskii A, Basso O. United States birth weight reference corrected for implausible gestational age estimates. Pediatrics 2014; 133:844-853.
3. Schieve LA, Devine O, Boyle CA, Petrini JR, Warner L. Estimation of the contribution of non-assisted reproductive technology ovulation stimulation fertility treatments to US singleton and multiple births. American Journal of Epidemiology 2009; 170:1396-1407.
