# Supplementary material for: Cancer in children born after frozen-thawed embryo transfer: A cohort study
Source: PLoS Med. 2022 Sep 1;19(9):e1004078. doi: 10.1371/journal.pmed.1004078 (PMC9436139; doi:10.1371/journal.pmed.1004078)
Supplement: Attachment — Submitted filename: Answer to referee 220521.docx [file pmed.1004078.s011.docx]

We thank editors and reviewers for valuable comments on our manuscript. Below follows detailed answers to Editors and reviewers.

We refer to pages and lines in the attached clean version of the manuscript.

Requests from the editors: 1) The Data Availability Statement (DAS) requires revision. For each data source used in your study: a) If the data are owned by a third party but freely available upon request, please note this and state the owner of the data set and contact information for data requests (web or email address). Note that a study author cannot be the contact person for the data. b) If the data are not freely available, please describe briefly the ethical, legal, or contractual restriction that prevents you from sharing it. Please also include an appropriate contact (web or email address) for inquiries (again, this cannot be a study author).

*Answer: We have revised The Data Availability Statement to the following: Data Availability Statement: Data cannot be shared publicly owing to restrictions by law. Data are stored at CoNARTaS folder at Statistics Denmark’s server, after receiving approvals by the Ethics Committees and registry keeping authorities in each country, as described in the following publication: Opdahl S, Henningsen AA, Bergh C, Gissler M, Romundstad LB, Petzold M, Tiitinen A, Wennerholm UB, Pinborg AB. Data Resource Profile: Committee of Nordic Assisted Reproductive Technology and Safety (CoNARTaS) cohort. Int J Epidemiol. 2020 Apr 1;49(2):365-366f. doi: 10.1093/ije/dyz228. Contact information for Statistics Denmark: Division of Research Services Statistics Denmark Sejrøgade 11 DK-2100 Copenhagen Denmark E-mail:forskningsservice@dst.dk Phone: +45 39 17 31 30.*

2) Abstract: a) Please ensure that all numbers presented in the abstract are present and identical to numbers presented in the main manuscript text.

*Answer: The numbers presented in abstract are correct. A minor correction is made on page 11, line 302 were aHR should be 1.65 and not 1.64 and on page 12, line 306 where the correct CI is “CI 1.33 to 4.12” and not “CI 1.33 to 4.11”.*

b) Please quantify the main results (please present both 95% CIs and p values).

*Answer: We have added p-values to our main results in Abstract and Result section..*

c) In the last sentence of the Abstract Methods and Findings section, please describe the main limitation(s) of the study's methodology.

*Answer: We have added the following sentence on page 3, line 64: “The main limitation is the number of children with cancer in the FET group.”*

3) Author summary – please include the main limitation(s) of the study

*Answer: We have added the following sentence on page 5, line 97. “The main limitation of this study is the number of children with cancer in the FET group.”*

4) Did your study have a prospective protocol or analysis plan? Please state this (either way) early in the Methods section. a) If a prospective analysis plan (from your funding proposal, IRB or other ethics committee submission, study protocol, or other planning document written before analyzing the data) was used in designing the study, please include the relevant prospectively written document with your revised manuscript as a Supporting Information file to be published alongside your study and cite it in the Methods section. A legend for this file should be included at the end of your manuscript. b) If no such document exists, please make sure that the Methods section transparently describes when analyses were planned, and when/why any data-driven changes to analyses took place. c) In either case, changes in the analysis-- including those made in response to peer review comments-- should be identified as such in the Methods section of the paper, with rationale.

*Answer:* *A prospective project plan dated 2020.04.28.11 has been added as Supporting Information and is now cited in Methods section, page 8, line 195 as follows:*

*“This study is reported as per the Strengthening the Reporting of Observational Studies in Epidemiology (STROBE) guideline (S1 Checklist). Our analyses were planned in advance of the research team accessing any data, and our study protocol is provided as supporting information (S1 Text). The CoNARTaS project is also registered in the ISRCTN registry (ISRCTN11780826).*

*The study plan has been modified to some parts and is presented as* *“Modifications to the original analysis plan for the presented analyses” (S1 Text).*

a) Thank you for providing the STROBE checklist. When completing the checklist, please use section and paragraph numbers, rather than page numbers.

*Answer: STROBE checklist has been revised (S1 Checklist).*

6) Statistical analysis a) Please include adjustment variables.

*Answer: Adjustment variables are described in Statistical analysis, page 234, line 9 but the text has been clarified to the following: “Adjustments were made for selected covariates. The variables included the following child and maternal characteristics: sex, plurality, calendar year of birth (continuous variable), country of birth, maternal age at delivery (continuous variable), and parity (nulliparous/parous). In a sensitivity analysis, we also included maternal smoking during pregnancy (yes/no) as a covariate. In an additional sensitivity analysis, maternal highest educational level achieved during the study period (low, medium, high) was included as a covariate.”*

b) Did you adjust for clustering at the parent level i.e., where 2 or more children are born via ART or spontaneous conception in a family

*Answer: No adjustment for clustering at the parent level was done. Please see answer to reviewer 3, point 8.*

7) Please provide p values in addition to 95% CIs in the main text and tables

*Answer: P-values in addition to 95% CIs in the main text and tables have been added.*

8) Please present and organize the Discussion as follows: a short, clear summary of the article's findings; what the study adds to existing research and where and why the results may differ from previous research; strengths and limitations of the study; implications and next steps for research, clinical practice, and/or public policy; one-paragraph conclusion.

*Answer: The Discussion has been reorganized according to your suggestion.*

9) In light of the study limitations, please avoid overstating your conclusions

*Answer: We have added the following: Abstract, page 4, line 69: “The results should be interpreted cautiously based on the limited number of events but the findings raise concerns considering the increasing use of FET, in particular freeze-all strategies without clear medical indications”.*

*Conclusion, page 15, line 925: “The results should be interpreted cautiously based on the limited number of events*.”

10) Please remove the ‘Data sharing sentence” and “Transparency declaration” from the end of the main text. In the event of publication, this information will be published as metadata based on your responses to the submission form.

*Answer: ´The ‘Data sharing sentence” and “Transparency declaration” have been removed in main text as suggested by reviewer.*

11) References: a) Please select the PLOS Medicine reference style in your citation manager. In-text reference call outs should be presented as follows noting the absence of spaces within the square brackets, e.g., "... services [1,2]." b) References should have six names before et al. For those with more than six names, please ensure that et al., is inserted after six names c) Please ensure that journal name abbreviations consistently match those found in the National Center for Biotechnology Information (NCBI) databases. https://journals.plos.org/plosmedicine/s/submission-guidelines#loc-references. d) Ref #1, 14, 30, 31, 32, 35, 36 are incomplete – please include the article titles. e) Please update ref #34 or delete if not published.

*Answer: Revision of references has been made as suggested. Reference #34 has been updated and replaced by: U.S. Department of Health and Human Services. National Institutes of Health. National Cancer Institute. International Classification of Childhood Cancer. ICCC Recode Third Edition ICD-O-3/IARC2017. Available from:* [*https://seer.cancer.gov/iccc/iccc-iarc-2017.html*](https://seer.cancer.gov/iccc/iccc-iarc-2017.html) *(Accessed May 16, 2022)*

**Comments from the reviewers:**

**Reviewer #1**: I  confine my remarks to statistical aspects of this paper.  The general approach is fine, but I do have some issues to resolve before I can recommend publication.

I especially commend the authors for building models based on science rather than on anything like forward, backward, or stepwise selection and for including sensitivity analyses.

1. One general issue is that the authors have an entire population, not a sample. Many statisticians (including me) feel that this makes inference (and, therefore, p values and CIs) irrelevant. There is no population to infer to. Others posit some "super population".  I don't find this persuasive (for one thing, how is your population a random sample from that?) but, if the authors want to do that, I won't forbid publication. But this needs to be dealt with.  One advantage of my approach is that it downplays p values altogether, which is a good thing because they are used far too much.

*Answer: Thank you for this interesting comment. It is correct that our population is not “a sample” of Nordic children but includes the entire population during the study period. Using such a scenario, p-values and CI would be irrelevant. However, we prefer to regard it as a sample of the world population of IVF children. Such an approach makes it possible for us to generalize our results to similar populations in other countries. This is of course important- we are not only interested in Nordic children. We also think that a major part of scientific papers in this field use such a strategy. Saying this we prefer to keep the study population as a sample.*

2.p. 9 line 210-211 -  Complete case analysis is only OK if the amount of missing data is either very small or missing completely at random.  I realize this would go in the Results section, but it could be mentioned here. (This is really a style issue, the editors may have a preference).  However, even in results, I only saw one mention of missing data, in the footnote to a table. So ... Please add something about this in the text.

*Answer: Missing data is presented in Supplementary Table 2 (now Table 1). In the main regression analysis adjustment was performed for sex, plurality, year of birth, country of birth, maternal age at birth and parity. For these variables the percentage of missing data is small (see Table 1). In two sensitivity analyses further adjustment was performed for maternal smoking or educational level. For both these variables missing data are substantial. Participants with missing data were excluded from these models. No imputations were made.*

*We have revised the page 9, line 234 to the following: “In the main regression analysis where adjustment was performed for sex, plurality, year of birth, country of birth, maternal age at birth and parity the percentage of missing data was small. In two sensitivity analyses further adjustment was performed for maternal smoking or educational level. For both these variables missing data were substantial. Participants with missing data were excluded from these models. No imputations were made.”*

3.Table 1 - there's nothing really wrong here, but it's a bit hard to read because of the number of columns and their organization. Are the number of children and number with cancer both needed? I think you can easily drop number with cancer, since you have the total and the %. Do you need both number of kids and PY?  And ... Maybe organize the columns by subject and then group rather than the reverse?  That is, have the three incident rates per 1000 children next to each other, and the incidence rate per PY next to each other?    Again, this is just to make it easier for the reader to make the most relevant comparisons.

or maybe table 1 could be organized like Table 3 with the sample sizes in the header?

*Answer: We prefer to keep absolute number of both children and number of cancers. However, we have reorganized the Table according to your suggestion A revised Table 1 is now included in the manuscript (now Table 2).*

4. Figure 1 - It would be better to combine the two panels and use a line for each group, rather than a series of bars.  Also, the labels on the y-axis could be made horizontal (but that is not a big deal).

*Answer: Figure 1 has been revised (now Figure 1 a,b,c) and the two panels are now combined. Figure legend for Figure 1 has been revised to the following. “Figure 1. Incidence of first cancer (all types) according to age and conception method among children born in Denmark (1994-2014), Finland (1990-2014), Norway (1984-2015), and Sweden (1985-2015) and followed from birth to 18 years.*

*a. Proportional distribution of age at first cancer diagnosis among spontaneously and ART-conceived children diagnosed with any cancer before age 18 years.*

*b. Age-specific hazard rates of first cancer among spontaneously and ART-conceived children diagnosed with any cancer.*

*The text is revised page 11, line 270. “The mean age at first cancer diagnosis was 6.0 years after ART and 6.8 years after spontaneous conception, and the distribution of age at first cancer diagnosis (Figure 1a) reflected the longer mean follow-up after spontaneous conception. Age-specific hazard rates were slightly higher among ART-conceived compared to spontaneously conceived children from approximately 5 to 12 years of age (Figure 1b), corresponding to unadjusted cumulative hazards which were similar up to about 6 years of age and diverged slightly thereafter (Figure 1c)”.*

5. Figure 2 - I would remove the lines for the CIs (especially considering my first point)  I might also remove the shading.  And same thing with the y-axis labels (This is easy to change in R, but I don't know about Stata).

*Answer: See answer to point 1 above. In accordance with this answer, we keep lines for CI and shading.*

6. Overall, though, a very good job.

*Answer: thank you!*


**Reviewer #2:**

1.I wrote a review of this analysis in December, 2021, when it was submitted to the Annals of Internal Medicine (see attachment). Although some small changes have been made, this version is essentially the same as that manuscript, therefore much of my original review and comments are still valid. The primary comment regarding this version is that the conclusion (in the abstract, and the Discussion) is that FET is associated with a higher risk of childhood cancer, yet the small sample size is the first comment in the limitations (page 12, line 305). Although the authors criticize the Hargreave paper (JAMA 2019; 322(22):2203-2210) for only having 14 cancer cases among their FET cohort, many of the analyses in this manuscript are based on very small sample sizes--Table 2 includes 5 cancer types based on <10 cancer cases, and 7 cancer types based on <20 cancer cases. My primary recommendation (in addition to those from my original review) would be to limit the
analyses to only all cancers and to leukemia.

*Answer: We have already, before submitting this paper to PlosMedicine revised this Table by excluding all adjusted HRs when the number of events was <10. We prefer however to keep figures for individual types of cancer for completeness and transparency. And maybe also for future meta-analyses. Our suggestion is thus to keep the table in its present form.*

2. This analysis should also be limited to residents of each of the study countries who received their ART treatment within their home country; the lack of emigration data for Finland is also problematic.

*Answer: Only residents in each country who received ART treatment within their home country are included.*

*We have added as limitation in Discussion page 13, line 390, the following sentences: “It was not possible to include Finland in the FET analysis due to missing information on ART method. Further, there was also lack on information on emigration from Finland”.*

Overall:

The authors present a detailed manuscript on the risk of childhood cancer by method of conception, using a population-based analysis spanning a 30-year period from four Nordic countries. This is an important area of research, as conception using assisted reproductive technology (ART) and in vitro fertilization (IVF) has risen dramatically worldwide since the first birth from this technology in 1978. This consortium of researchers (the CoNARTaS group [Committee of Nordic Assisted Reproductive Technology and Safety]) have been leaders in pooling population-based data to answer important questions about maternal and child health after assisted reproduction.

3. Upon deeper inspection, there are numerous gaps and inconsistencies in the data between the four countries in this study including the sources, types, and years reported. Although the authors refer the reader to other references for detail regarding the data from each country (References 24-30, and 42), it would be more relevant and transparent to include a table within this manuscript detailing the birth, ART, IVF, birth defects, and cancer data from each country, and the sources and standards for each type of data (self-reported by the mother at delivery versus by the physician or midwife, reported by the physician to a registry using standardized definitions). In addition, the data should be limited to residents of each country who received their ART or IVF treatment within their own country.

*Answer: A Supplementary Table S1 according to your suggestion has been added to the manuscript. Data only include residents of each country who received ART and delivered within their own country.*

4. Limitations:

-The authors rely on several references (References 24-30 and 42, and the Marsál et al reference in the Supplemental Tables section) to describe important measures used in this study; this requires reading through those additional references to understand what was used. The birthweight reference for classifying infants as small-for-gestation (SGA) (Marsál et al, 1996), which is based on only 86 uncomplicated pregnancies, defines SGA as -2 SD below the mean, although the mean for each gestation (days) is not given; there is no definition for LGA in this reference, although the authors of this analysis reference Marsál et al for both SGA and LGA in their tables (Tables S2, S3). A preferable measure to define both SGA and LGA is birthweight z-score, with cutoffs of <1.28 and >1.28, respectively. There are several methods available to calculate birthweight z-scores, including the Fetal Medicine Foundation weight charts^[1]^ (which are also available online at <https://fetalmedicine.org/research/assess/bw>), and the US reference by Talge et al^[2]^, which is based on 7.8 million births in 2009-10 from 22-44 weeks gestation; means and standard deviations for birthweights by infant sex are given for each week, from which z-scores may be calculated.

Answer: *A new Supplementary Table S1 according to your suggestion with information on measures used in this study has been added to the manuscript (please also see answer to comment 3).*

*Concerning the birth weight reference Marsál et al. (1996) we agree with the reviewer that an appropriate reference curve is critical and this reference may not be optimal. A new Swedish reference curve with ultrasound estimated fetal growth (EFW) has recently been published and is currently being validated (Lindström et al., Sci Rep. 2021 Jun 14;11(1):12464. There are limitations of using observed birthweight instead of EFW according to gestational age from intrauterine (ultrasound) measurements of ongoing, presumably healthy pregnancies. As preterm birth often results from pregnancy complications that can affect fetal growth, the observed birthweights in preterm deliveries are not representative of the normal fetal weight distribution for healthy pregnancies at a given gestational age. We therefore prefer to have a reference based on ultrasound and not on observed birth weight. Multiple international standards of EFW have been presented and there is still debate on which of these charts should be used. Large efforts have been made to create a standard that can be universally adopted* *based on the principle that infants worldwide should have the same growth potential, however, difficulties with implementing international standards have been highlighted (Vieira et al., PLoS Med. 2019 Sep 20;16(9):e1002902). The study by Marsáll et al.(1996) was based on pregnancies among Danish and Swedish women during the first part of our study period and are currently the reference used with a +/- 22% limit in clinical practice in Sweden (Lindström et al., 2021). Despite a small number of participants, the estimated growth patterns are very similar to those of a larger, more recent Norwegian population (Johnsen et al., Acta Obstet Gynecol Scand. 2006;85(3):286-97).*

*The reviewer is correct that using the z-score with cutoffs of <1.28 and >1.28, (or <10th and >90th percentile) respectively, of observed birthweights is a commonly used definition of SGA and LGA. The use of ±22% as the limits for LGA and SGA, corresponds approximately to the 2.3 and 97.7 percentiles. Using the 10th and 90th percentile from an intrauterine standard would increase sensitivity, but at the cost of specificity by including a large number of constitutionally small or large fetuses/neonates as SGA or LGA (Alberry et al., Arch Dis Child Fetal Neonatal Ed. 2007;92(1):F62-7.)*

*We have not made any changes to use of reference curve and our definition of SGA and LGA in the paper and hope the reviewer accepts our rationale for this.*

5. Specific Comments:

-Throughout this manuscript, the authors state that the study population is based on births in 1984-2015 in four Nordic countries (including the sentence in the Methods section of the Abstract, “This cohort study included 171,774 children born after use of assisted reproductive technology (ART) and 7,772,474 children born after spontaneous conception, ***representing all children born between 1984 and 2015 in four Nordic countries***). In evaluating this statement, the study population actually includes 1994-2014 births in Denmark, 1990-2014 births in Finland, 1984-2015 births in Norway, and 1985-2015 births in Sweden.

*Answer: This is correct. However, this comment was already corrected in the PlosMedicine submitted version.*

6. -The description of the study populations by each country is given in Table S2; this description should have been the first table in this paper, and not put in the supplementary section. In addition, the racial and ethnic composition of the study population is not given, nor are these factors included in any of the regression models. This is an important limitation, as there are well-establish differences in childhood cancer by these two factors.

*Answer: We have changed order of Tables. Table S2 is now Table 1. Variables as ethnicity and race are not allowed to register in the Nordic countries. For Sweden country of birth was available in a previous CoNARTaS publication (Norrman et al. Hum Reprod. 2018 Oct 1;33(10):1948-1959) and the following was reported for country of birth for the mothers :*

ART	SC

Sweden	88.8%	87.0%

Nordic countries outside Sweden	3.0%	3.3%

Europe	4.5%	4.1%

Africa	0.5%	1.0%

Asia	2.5%	3.6%

Other	0.8%	1.1%

*It has been reported that non-white children and young adults might have a lower rate of several childhood cancers (Marcotte EL, et al. Cancer. 2021 Oct 1;127(19):3651-3663). The percent of non-white in our population is probably very low. Further, we have no possibility to adjust for race/ethnicity.*

*We have added in the Discussion page 14, line392 as a limitation: “Adjustment for race/ethnicity was not possible since registration on race/ethnicity is not allowed in the Nordic countries. It has been reported that non-white children and young adults might have lower rates of some childhood cancers [50]. The percentage of mother's country of birth being outside the Nordic countries was however low and similar in ART and spontaneous conception in an earlier publication from CoNARTaS [51]”.*

-The ART group represents a combination of both fresh and frozen embryo transfers, with cryopreservation reported for varying periods of time (1994-2014 for Denmark, 1988-2015 for Norway, 1990-2015 for Sweden), or not at all (Finland), and with cryopreservation methods (slow-freezing or vitrification) only reported for 2009-2014 in Denmark and 2006-2015 for Sweden; data on the use of ICSI is only available for 1994-2014 in Denmark, 1996-2015 in Norway, and 1992-2015 in Sweden; and data on culture duration (day of embryo transfer) only available for 1994-2014 in Denmark, 2011-2015 in Norway, and 1985-2015 in Sweden (Table 3 in Reference 24, Opdahl et al, Int J Epidemiology 2019; 1-8).

7. -The spontaneously-conceived births include women who were treated with non-IVF ART (ovulation induction and intrauterine insemination), resulting in some degree of misclassification. A new subgroup could be generated (non IVF-ART) for 2007-2015 births in Denmark, for 2016-17 births in Finland; it is unclear if this data is available in Norway and Sweden.

*Answer: It is correct that women treated with ovulation induction and intrauterine insemination (IUI) are included in the spontaneously conceived group and thereby resulting in some degree of misclassification. For Denmark today the number of deliveries after IUI is considerable, about half the number of ART cycles, similar for Finland today about a fifth the number of ART cycles. For Sweden and Norway it is around 5% or less. For the total study period non-ART fertility treatment probably constitute less than 1% of the spontaneous conception (SC) population. Most IUI use natural cycles and stimulated cycles only for anovulatory women. It is unfortunately not possible for us to in the present cohort separate these nonART treatment cycles from SC population and perform sub analyses as suggested by reviewer. In Hargreave et al. (JAMA 2019;322: 2203-2210), such subgroup analyses were performed for different kind of hormonal stimulation and for any kind of hormonal stimulation, resulting in adjusted HR close to unit (SC children as reference). We have more clearly stressed this misclassification as a limitation in our manuscript. If any, this misclassification will attenuate the association, not strengthen.*

*In Discussion, page 14, line 400 we have revised to: “We were not able to exclude other medically assisted reproduction methods such as intrauterine insemination or ovulation induction from spontaneously conceived children. Although such cycles today, at least in Denmark are substantial, they only accounted for a small proportion of the spontaneous conception cohort. This misclassification might have attenuated the associations.”*

8. -Medical tourism is frequent, particularly in Denmark. ART conception is included in the Medical Birth Registry in Norway, based on information provided by the mother at delivery “…allowing for registration of children conceived after ART treatment abroad” [Opdahl et al, Int J Epidemiology 2020; 49:365-366f].

*Answer: Fertility tourism, defined as patients going abroad for fertility treatment and delivering in their home countries or patients coming from abroad and performing ART in a Nordic country and then delivering in their home country are not included in the present ART population. Although such medical tourism is rather frequent today in Denmark it was much less the years this study includes. Today around 900 IVF/ICSI cycles and a couple of thousands IUI cycles are performed in Denmark using fertility care tourism, meaning that patients are coming from abroad, doing treatment in Denmark and then deliver in their home countries. Such patients can of obvious reasons not be included in the Danish population of this study. And they cannot either be identified in the other Nordic registers for certainty. Tourism going the other way are neither possible to correctly identify. We have added the following sentence in the Discussion, page 15, line 405.*

*“In the present study only patients performing ART and delivering in their home countries are included. Although fertility tourism, meaning that patients go abroad or coming from abroad for fertility treatment today is rather common in some Nordic countries, this was uncommon during the study period. Such cycles are further impossible to correctly identify.“*

9. -The CoNARTaS description of birth defects registries (from Table 2 in Reference 24) in the four countries included in this analysis indicate that Finland has maintained this type of registry for 1990-2014 births, Sweden has done so for 1964-2015 births, while reporting birth defects has been through the Medical Birth Registry in Norway, and is not available for births in Denmark.

-Major birth defects should be separated into chromosomal, nonchromosomal, and the presence of both types within the same child, as it is well-established that children with chromosomal birth defects are at much greater risk for childhood cancer.

*Answer: Description on birth defects registration is now included in the new Supplementary Table S1*

*We have interpreted the reviewer that data on 1. any major birth defects (with or without chromosomal defects 2. major non-chromosomal birth defects and 3. chromosomal defects (with or without other major birth defects) should be reported which is similar as in the article by Luke et al. (Luke B Assessment of Birth Defects and Cancer Risk in Children Conceived via In Vitro Fertilization in the US. JAMA Netw Open. 2020 Oct 1;3(10):e2022927, Table 2). This information is now included in Table 1 (previous Table S2), and in Table S3. We have also added the rate of chromosomal aberrations among leukemias. Please see answer to reviewer 4, point 4.*

10. -Multifetal pregnancies are not divided into twins, triplets, and higher-order births; this should be specified.

*Answer: Yes, multifetal pregnancies are divided in twins, triplets and high order pregnancies, see Table S2 (now Table 1).*

11. -There are too few cancer cases to present models for frozen embryo transfer, and the models are missing critical factors such as infertility diagnoses, the use of assisted hatching and ICSI, the day of embryo transfer (days in culture, as cleavage-stage versus blastocyst-stage), as well as maternal race and ethnicity (as stated earlier). Most of the supplemental tables and models should be eliminated (Table 2 results of the 12 cancer types includes 4 models based on <10 cases of cancer in ART children and 8 models based on <30 cases among ART children).

*Answer: See reviewer 2, point 1. Further, in Table S5 which we assume you more specifically refer to, we had already revised this table by excluding adjusted HR.*

*Infertility diagnoses were not included due to a lot of missing data. Assisted hatching has almost not been used in the Nordic countries. The proportion of ICSI was presented in S3 Table and the percentage of cleavage stage embryos and blastocyst are now added in the S3 Table. Concerning ethnicity-see reviewer 2, point 6.*

*Due to the limited number of events it would not be possible to adjust for more variables then what has been done.*

*Please also see answer to reviewer 3, point 6 concerning selection of possible confounders. The models rely on earlier knowledge (Reigstad et al., Pediatrics. 2016 Mar;137(3):e20152061). There is also an increased risk of over-adjustment if building larger models.*

12. -The authors minimize acknowledgement of the limitations of their study, citing that it is observational data, and that non-IVF ART (intrauterine insemination or ovulation induction) could not be excluded, but only accounted for a “very small proportion of the spontaneous conception cohort.” As stated above, this data is available for Denmark, and may account for a much larger proportion of births than stated. The Center for Disease Control estimated that non-IVF ART accounts for four-times as many IVF-conceived births on an annual basis.^[3]^

*Answer: Please see answer above, reviewer 2, point 7. We have more clearly stated this limitation.*

Minor Comments:

Spelling of:

New Zealand (line 72, page 4) *This was corrected in the PlosMedicine submission*

Macrosomia (Supplemental tables) *This was corrected in the PlosMedicine submission*

Carry over headings when tables cross multiple pages (Table S2)- *Tables have been revised, however, unfortunately we did not manage to do this with Table 1.*

**Reviewer #3:** This is a very important study, dealing with an exposure that is relatively new, still it is becoming more prevalent. The study is based on a very large sample size, which is a major advantage, as mentioned by the authors, with a long follow-up time, which is suitable to study such outcomes.
I do have a few suggestions and comments:
1.In the Abstract- Please add the rate of ART in this cohort (not only the n`s).

*Answer: We have added the rate of ART, 2.2% in Abstract, line 45:*

*“The study included 7 944 248 children, out of whom 171 774 children were born after use of ART (2.2%) and 7 772 474 children were born after spontaneous conception, representing all children born 1994-2014 in Denmark, 1990-2014 in Finland, 1984-2015 in Norway, and 1985-2015 in Sweden”.*

2. In the Abstract- please explain why there are 2 follow-up periods mentioned.

*Answer: The different countries have different routines when registry data are made available for research.*

3. Please add in the Abstract how many cases were diagnosed among the fresh embryos, and be consistent with the reporting style.

*Answer: In Abstract we have presented the total number of cancers in all ART, 329 cases. Further, the number of cancers in FET, 48 cases. However, in the FET analysis only data from Denmark, Norway and Sweden is included since the ART methods cannot be separated in the Finnish data. This is clearly stated in the Method section. In the three countries Denmark, Norway, and Sweden 227 cases of cancer occurred in the fresh embryo group. These numbers are presented in Results in the manuscript and the situation in Finland is explained. To present this number also in Abstract would need a rather long explanation about Finland, otherwise it will be confusing for the reader. We thus, in Abstract chose to only give numbers for (known) FET and for all ART.*

4. In the Abstract- Obviously the models did not adjust for both macrosomia and birthweight, please clarify.
In the abstract- "all children born 1994-2014…" should be- all children born between the years ….
The sentence in line#120 ("In many countries, the number of FET-conceived children has now exceeded the number born after fresh embryo transfer.") should be transferred to line#104.

*Answer: We did not adjust for both macrosomia and birth weight in the same model since these variables are not independent. In another model we adjusted for birth weight as a continuous mode. See page 10, line 239.*

*We have revised Abstract line 60: Adjustment either for macrosomia, birthweight, or major birth defects attenuated the association marginally.*

*We have revised Abstract line 46 and added “born between the years….”*

*We have revised the Introduction to the following, page 6, line 127: “Currently, up to 7.9% of children in Europe and 5.1% in the United States are born after ART, making health of children born after ART a topic of public health importance [14,15].*

*In many countries, the number of FET-conceived children has now exceeded the number born after fresh embryo transfer [1,16]”.*

5. The following sentence is unclear: "Cancer in childhood is heterogeneous especially concerning morphology" , line#124. What does it mean cancer is  heterogeneous? In what way?

*Answer: We have rephrased and clarified this in the Introduction, page 6, line 131: “Childhood cancer includes a wide array of diagnoses, some of them very rare. Often the diagnoses are seen only in children but also cancer diseases common in adults occur. Leukaemia is the most common neoplasm followed by various forms of tumours in the central nervous system”.*

6. In the Methods section- How were the co-variables in the models selected?
In the results section- lines#250-262, several multivariable models are mentioned- what were included in these models?

*Answer: Selection of confounders was based on medical knowledge and previous studies. We searched literature for identification of confounders. The following has been added in the Method section, in Statistical analysis, page 9, line 225*

*“Selection of covariates was based on medical knowledge and previous studies. We searched literature for identification of covariates [ref Reigstad MM, Larsen IK, Myklebust TÅ, Robsahm TE, Oldereid NB, Brinton LA, Storeng R. Risk of Cancer in Children Conceived by Assisted Reproductive Technology. Pediatrics. 2016 Mar;137(3):e20152061. doi: 10.1542/peds.2015-2061. Epub 2016 Feb 4. PMID: 26908669; PMCID: PMC5178823]”.*

7. What the authors performed is not a sensitivity analysis in sub-populations, rather they tested several models.
What does it add to show the results by country?

*Answer: To show results by country is regarded as a check for homogeneity between countries and robustness of the paper.*

8. Indeed, as the authors mentioned- there is a possibility of selection bias, as the best quality embryos may have been selected for fresh embryo transfer. The authors should consider using propensity scoring analysis, to possibly account for this selection bias.

*Answer: We have no information on -variables associated with embryo morphology available, thus making propensity score not possible. Further, “FET” from a clinical view is regarded a combination of the freezing/thawing procedure and selection of embryos for FET and should be assessed as this combination.*

Did the authors account for siblings in the cohort, and how?

*Answer: We did not adjust the analysis for potential clustering among siblings since we had a very large number of clusters and the cluster sizes were normally below three. This means that the clustering effect will be very limited, and given that frailty models are relatively more unstable we preferred to not adjust for clustering.*

**Reviewer #4:** This is a very well-written manuscript using a robust population-based approach to evaluate associations between frozen-thawed embryo transfer (FET) and cancer in children. The analyses were comprehensive and clear, and the findings are notable. I do have a few questions for the authors to consider.

1. Methods: While I have no objections to the choice of covariates for the analysis, I was curious about the rationale for the selection of certain variables. Was this done based on previous assessments, the construction of a DAG, or some other strategy? I think providing some rationale for variable selection in the Methods section would be helpful.

*Answer:* *Selection of confounders was based on medical knowledge and previous studies*. *Please see answer to reviewer 3, point 6.*

2. Methods: I appreciate that the authors considered the role of birthweight and birth defects on their results. I have two questions related to this. First, how was information on birth defects obtained? Were similar linkages done using congenital anomaly registries, or was this information obtained from birth records. If the latter, this is a potential limitation for this analysis as birth records are neither sensitive nor specific for the assessment of birth defects (see PMID: 27859434). I think this point must be addressed. Second, if the authors propose that these conditions could be important in the potential association between FET and cancer in children, it seems as if they would be on the causal pathway rather than factors that need to be considered as confounders. I think even describing the prevalence of macrosomia and birth defects in those exposed to FET with and without cancer would be interesting. Ultimately, can the authors justify this approach (i.e., thinking
of phenotypes on the causal pathway as confounders)?

*Answer: Please see Supplementary Table S1 for the question concerning where information has been obtained. Registration of birth defects in the Medical Birth Registries are based upon pediatric examination of all newborn in the Nordic countries before discharge. We agree that some birth defects may be diagnosed after discharge. We therefore also used national patient registries for information on birth defects, in all countries except Norway. The follow up time is at least one year.*

*Concerning separating FET characteristics macrosomia, LGA and major defects and chromosomal aberrations, we have added the following sentence in Results page 12, line 306: ”In the FET group, the rates of macrosomia, LGA and major birth defects were: 31.3% vs 19.4%, 10.6% vs 5.6%, 6.3% vs 4.3%, in the cancer and no cancer groups”.*

3. Methods: Use of the ICCC-3 for cancer classification is a strength. Can the authors confirm if these classifications were done for older years by the respective cancer registries (I don't think the ICCC-3 was used until the 2000s) - or did the authors use an algorithm to code these cancers? Either way, can the authors include this information in the Methods?

*Answer: We have clarified this in the Method section, page 8, line 174:* *“The primary outcomes were any cancer before age 18 years after use of any ART and specifically after FET. Secondary outcomes were cancer diagnosis groups according to the International Classification of Childhood Cancer (ICCC-3) [34]. The ICCC-3 is based on the WHO classification system for cancer morphology and allows comparison of broad categories of neoplasms in continuity with previous classifications [35]). In ICCC-3, the diagnoses are grouped into 12 main categories according to the morphology code, the topographic code and the behavior of the tumor, i.e., benign or malignant (Supplementary Appendix S2 Table). We grouped all patients into ICCC-3 categories. The 12 groups each include a defined set of morphology codes and occasionally the additional use of topography codes, was used.  In older patients topography codes according to the International Statistical Classification of Diseases and Related Health problems were transferred to the latest version, ICD-10, by an algorithm used by the cancer registries. ICCC-3 only groups tumors with a malignant diagnosis except for tumors located in the CNS. Consequently, other benign or borderline tumors were not included in this report. Although there are discrepancies, due mainly to different traditions in cancer registration between the countries [27,33], pooling of data was possible because all use the WHO classification system [35].*

4. Results: The finding related to FET and leukemia is interesting. To that point, can the authors explain this result a bit more? For example, were these cases all ALL or AML? As trisomy 21 is a strong risk factor for pediatric leukemia, were any of these children with leukemia also diagnosed with trisomy 21? I know the authors adjusted for birth defects, but I think this particular congenital anomaly should be considered.

*Answer: Thank you for your interesting questions. Concerning the proportions of AML and ALL , this is not possible to separate in the present cohort but will be done in future analysis.*

*The following frequencies of all chromosomal aberrations among leukemias were observed.:*

*Chromosomal aberrations among leukemias:*

*ART: 5/111= 4.5%*

*SC: 109/4921=(2.2%)*

*FET: 0/23*

*Fresh 3/75 (4.0%)*

*SC: 86/3952-(2.2%)*

*We have added the following sentences in Results, page11, line 282: The rates of any chromosomal aberration among children with leukemia were 4.5% in ART and 2.2% in the spontaneous conception group.*

*and page 12, line 317: The rates of any chromosomal aberration among children with leukemia were 0% in the FET, 4.0% in the fresh and 2.2% in the spontaneous conception group*

Any attachments provided with reviews can be seen via the following link:
<https://www.editorialmanager.com/pmedicine/l.asp?i=773494&l=63I8AEL0>

__________________________________________________
In compliance with data protection regulations, you may request that we remove your personal registration details at any time.  (Use the following URL: <https://www.editorialmanager.com/pmedicine/login.asp?a=r)>. Please contact the publication office if you have any questions.Review of Cancer in Children Born After Frozen-Thawed Embryo Transfer: A Cohort Study, by Sargisian et al.

1. Nicolaides KH, Wright D, Syngelaki A, Wright A, Akolekar R. Fetal Medicine Foundation fetal and neonatal population weight charts. Ultrasound in Obstetrics and Gynecology 2018, pp 1-8. Doi: 10.1002/uog.19073.
2. Talge NM, Mudd LM, Sikorskii A, Basso O. United States birth weight reference corrected for implausible gestational age estimates. Pediatrics 2014; 133:844-853.
3. Schieve LA, Devine O, Boyle CA, Petrini JR, Warner L. Estimation of the contribution of non-assisted reproductive technology ovulation stimulation fertility treatments to US singleton and multiple births. American Journal of Epidemiology 2009; 170:1396-1407.
