# Supplementary material for: Cancer in children born after frozen-thawed embryo transfer: A cohort study
Source: PLoS Med. 2022 Sep 1;19(9):e1004078. doi: 10.1371/journal.pmed.1004078 (PMC9436139; doi:10.1371/journal.pmed.1004078)
Supplement: Attachment — Submitted filename: Answer to reviewer_PLOSMED rev2_220714.docx [file pmed.1004078.s012.docx]

We wish to thank all the editors and reviewers for comments and constructive feedback. We have addressed the questions point by point below and we hope we have answered the questions appropriately.

-----------------------------------------------------------
Requests from the editors:

1.      Please pay particular attention to reviewer #3’s remaining concerns regarding your statistical approach. We will not be able to proceed until you respond to this.

Answer: We have responded to reviewer #3 concerning the statistical approach.

2.      Please report p <0.001 instead of p <0.01

Answer: We report p=0.005 and p=0.009 instead of p<0.01 and hope that we have understood this correctly.

3.      Abstract
a.      Change subheading to “Methods and findings” not “Method and findings”

Answer: We have changed the subheading to ”Methods and findings”

b.      Last sentence of the “Methods and findings” should read, “The main limitation of this study is the small number of children with…” Please update the author summary as well.

Answer: We have revised the last sentence in Methods and findings to ”The main limitation of this study is the small number of children” and updated the author summary as well: ”The main limitation of this study is the small number of children with cancer in the FET group”.

.
4.      Please remove subheadings from the Discussion section

Answer: We have removed the subheadings from the Discussion section

In addition, a new reference is added, published after our first submission: *11. Maheshwari A, Bell JL, Bhide P, Brison D, Child T, Chong HY et al. Elective freezing of embryos versus fresh embryo transfer in IVF: a multicentre randomized controlled trial in the UK (E-Freeze). Hum Reprod. 2022 Mar 1;37(3):476-487. doi: 10.1093/humrep/deab279. PMID: 34999830*

Comments from the reviewers:

Reviewer #2: The authors have been responsive to most of the issues raised in the review. Two issues remain: the use of Marsal et al, 1996 birthweight reference to characterize the study children as SGA or LGA; and Cox proportional hazards models based on <5 FET children. For the birthweight reference, since neither SGA nor LGA is used in the models (with macrosomia and LBW used instead), the authors should eliminate the SGA and LGA data and related text, and in future analyses, use a better reference when generating these cut-offs. For the models using <5 FET children in the 7 out of 9 cancer types (Table S5), I still feel strongly than even giving the crude HRs is somewhat misleading, although the authors have excluded the adjusted HRs. I understand their desire to include absolute numbers for the possible inclusion in future meta-analyses.  In closing, I want to commend the authors for this ambitious study, and for its contribution to the field.

Answer:

We have deleted the Marsal reference and SGA and LGA data as child characteristics in Table 1 and in S3 Table and in the text, page 11, line 278.

We have deleted the crude HRs and adjusted HRs in Table 3 and in S5 Table if the number of ART children is <10.

Reviewer #3: While most of my comments have been addressed in the revised submission, I still have concerns about the selection of variables in the multivariable analysis.
Selecting variables based on the literature is not the correct and may not be relevant in this study population. The selection should be based on testing the confounding effect,
model fit, and testing possible collinearity between co-variables.
This is not mentioned and the methods not detailed.
Additionally, lack of data regarding sperm morphology does not make propensity score analysis irrelevant or impossible, and still I recommend adding this analysis to strengthen your findings.

Answer:

**Selection of covariates:**

Several ways exist to select covariates. Previous studies on cancer risk after ART conception selected covariates in different ways: Reigstad et al. (Pediatrics 2016 PMID: 26908669) and Spector et al. (JAMA Pediatr. 2019, PMID: 30933244;) both used literature and with references. Other authors (Hargreave et al., JAMA. 2019, PMID: 31821431 and Spaan et al. Hum Reprod 2019, PMID: 30715305) both defined covariates as factors changing the HR for ART by 10% or more.

Further, <https://www.ncbi.nlm.nih.gov/books/NBK126194/> strongly recommend, as first approach, to select variables on the basis of background knowledge about the relationship of the variable to treatment and outcome. The second approach relies primarily on statistical associations to select variables.

We would like to avoid a data driven approach for the selection of covariates to not run into an explorative study design. As pointed out in the paper the covariate selection is primarily based on previous observations and knowledge in the area. Furthermore, all selected covariates, except for plurality had a *p*-value<0.25 in main analyses, supporting them as possible covariates.

Collinearity was assessed via the post-estimation command estat VCE in Stata giving the covariances/correlations between the different covariates in the Cox proportional hazards model. The following correlations were the maximum for the main analysis (all cancer):

- ART vs SC: ART had a correlation with plurality of -0.22, and parity had a correlation with age at birth at 0.36.

- FET vs fresh embryo transfer: FET had a correlation with parity of 0.11, and parity had a correlation with age at birth at 0.24.

- FET vs SC: FET had a correlation with plurality of -0.07, and parity had a correlation with age at birth at 0.37.

These correlations are expected due to logical relations. Correlations for the other covariates were below 0.05. We thus believe we that we do not have any major issues with multicollinearity in our analysis.

The following paragraph has been revised and more details have been added in Method section, pages 9-10, line 222-261:

*We estimated crude and adjusted hazard ratios (HRs) with 95% confidence intervals (CIs). The significance level was set to 5%. A number of <10 events in any group was considered too small to calculate a stable estimate. Adjustments were made for selected covariates. Selection of covariates was primarily based on medical knowledge and previous studies. We searched literature for identification of covariates [38]. The variables included the following child and maternal characteristics: calendar year of birth (continuous variable), country of birth (Denmark, Finland, Norway, Sweden), maternal age at delivery (continuous variable), parity (nulliparous/parous), sex, and plurality (singletons/multiples). Calendar year at birth and country of birth both influence cancer incidence as well as likeliness of having been conceived by ART. Both maternal age and birth order have been shown to be associated with cancer in offspring [39, 40] and are also associated with ART (ART mothers are older and of lower parity than spontaneous conception mothers). Risk of certain cancers is different among males and females [41] and some ART methods (transfer of blastocysts) may alter the sex ratio [42] and therefore sex was included as a covariate. Furthermore, an association with multiple birth and cancer (leukemia) has been found [43] and multiple birth is more common after ART, and plurality was therefore also included as a covariate.*

*In a sensitivity analysis, we also included maternal smoking during pregnancy (yes/no) as a covariate. In an additional sensitivity analysis, maternal highest educational level achieved during the study period (low, medium, high) was included as a covariate [44]. This analysis included data from Denmark, Finland, and Sweden because data on education were not available from Norway.*

*In the main regression analysis where adjustment was performed for year of birth, country of birth, maternal age at birth, parity, sex, and plurality the percentage of missing data was small. In the sensitivity analyses where adjustment was performed for maternal smoking or educational level missing data for these variables were substantial. Participants with missing data were excluded from these models. No imputations were made.*

*Macrosomia and major birth defects have been associated with childhood cancer [45-47] and are also associated with ART [23, 48]. To investigate macrosomia and major birth defects, as possible mechanisms of an increased risk of cancer in children born after FET, separate exploratory analyses were performed with additional adjustment for macrosomia (yes/no) and major birth defects (yes/no). A similar analysis was also performed with birthweight as a continuous variable. Finally, as an indicator of embryo quality, we additionally adjusted for embryo stage i.e. cleavage stage or blastocyst in a separate exploratory analysis comparing conception after FET and fresh embryo transfer.*

*Collinearity was assessed via the post-estimation command estat variance–covariance matrix of the estimators (VCE) in Stata giving the covariances/correlations between the different covariates in the Cox proportional hazards model. No major issues with multicollinearity were identified in our analyses.*

*The proportional hazards assumption was tested with Schoenfeld residuals, and there were no clear violations. All analyses were performed in Stata, version 16.*

**Embryo morphology and propensity score**

The original concern from the reviewer was that there could be residual confounding between fresh and frozen embryo transfers due to differences in embryo quality, assuming that the embryo with the highest quality would be transferred first, as a fresh embryo. However, one could also argue the opposite, that embryos surviving additional culture to blastocyst stage and the subsequent vitrification and warming, would have a higher quality. The rate of blastocysts was higher in the FET compared to the fresh transfer group. Survival to blastocyst stage can be seen as a marker of embryo quality. Numerous studies have shown that the live birth rate is significantly higher per embryo transfer for blastocyst compared to cleavage stage embryo transfer (Glujovsky et al. Cleavage stage versus blastocyst stage embryo transfer in assisted reproductive technology. Cochrane Database Syst Rev 2016; CD002118, Fernandez-Shaw S et al. Ongoing and cumulative pregnancy rate after cleavage-stage versus blastocyst stage embryo transfer using vitrification for cryopreservation: impact of age on the results. J Assist Reprod Genet 2015;32: 177–184). Further, in the fresh embryo transfer group there is a certain proportion of poor or less good quality cleavage stage embryos transferred, since in patients producing only one embryo that embryo will be transferred, more or less independent of embryo quality while in the FET group only good quality embryos are frozen.

We have now added embryo stage as an indicator of embryo quality i.e. cleavage stage or blastocyst as a covariate in an additional analysis, comparing conception after FET and fresh embryo transfer; Table 4, S5 Table, and in the Result section page 12, line 329 and page 13, line 339). We have no variable indicating embryo quality for cleavage stage embryos in the registry. Adjustment for embryo stage slightly strengthened the association between FET and cancer.

We believe that this solution will address the reviewer’s concern better than by introducing propensity scores, because we have very little pre-treatment information in our data that can help predict whether an ART-conceived child was conceived through fresh or frozen treatment. However, a very large part of the cohort of FET patients are patients that primarily have done a fresh cycle, not resulting in a live birth and then have used cryopreserved surplus embryos from the same oocyte retrieval. That has been the tradition in the Nordic countries and the “freeze-all” concept (i.e. freezing of all embryos from a treatment cycle and no fresh embryo transfer) was almost not used at all during the study period (1985 to 2015). Please see attached graph where the number of freeze all cycles is extremely few during the actual study period (1985 to 2015) compared to around 10 000 fresh embryo transfers performed yearly in Sweden, even though an increase is noticed during later years. A similar situation has occurred in the other Nordic countries.

We apologize for the Figure text in Swedish. The graph shows the number of “freeze-all” per year with national data from Sweden from 2010 to 2021. The number of fresh transfers per year is around 10 000. Green bars indicate ”freeze-all” due to OHSS and yellow bars indicate ”freeze-all” due to other causes.

The year 2014 it was zero “freeze-all” in both groups (OHSS risk and other).

Saying this, the FET population probably represents more good prognosis patients since it was possible to freeze embryos in the same cycle in addition to the fresh transfer.

In summary, we would argue that both at the patient and at the embryo level, the FET group represents a somewhat “healthier” group than the fresh embryo transfer group.

We have slightly modified the text in the Discussion section, page 15, line 430

*In addition, more FET pregnancies were conceived after transfer of blastocysts which are considered to have higher quality than cleavage stage embryos. When adjusting for embryo stage as an indicator of embryo quality, the association between FET and cancer slightly strengthened. Furthermore, a vast majority of FET cycles were performed after a failed fresh cycle from the same oocyte retrieval and cryopreservation of surplus embryos while the freeze-all concept was hardly used* *in the Nordic countries during the study period. In fact, the FET population probably represents more good prognosis patients since it was possible to freeze embryos in the same cycle in addition to the fresh embryo transfer.*
